# Relationship between biomarkers and stroke risk in sickle cell disease odds in an Amazon folk

**DOI:** 10.1590/1678-4685-GMB-2025-0107

**Published:** 2026-07-10

**Authors:** Greice de L. Cardoso-Costa, Fernando Mendes Paschoal, Bruno Miranda, Alessandro Cardoso Rodrigues, Giovanna C. Cavalcante, Caio S. Silva, Saide Maria Sarmento Trindade, Sergio Antônio Batista dos Santos, Milene Moraes Raiol, Cristina Maria Duarte Valente, Leticia Dias Martins, Nelson Machado da Silva de Lima, Joelma Karin Sagica Fernandes Paschoal, Fernanda Andreza P. L. Figueiredo, Sidney Santos, Eric Homero Albuquerque Paschoal, Ândrea Ribeiro-dos-Santos

**Affiliations:** 1 Universidade Federal do Pará, Instituto de Ciências Biológicas, Laboratório de Genética Humana e Médica, Belém, PA, Brazil.; 2 Instituto AVC da Amazônia, Belém, PA, Brazil.; 3 Fundação Centro de Hemoterapia e Hematologia do Pará (HEMOPA), Belém, PA, Brazil.

**Keywords:** Sickle cell anemia, stroke, amazon, biomarkers, genetic

## Abstract

Sickle cell disease (SCD) is defined by important heterogeneity between patients, but this clinical presentation mutability is not well understood. Therefore, this immense phenotypic variability calls for research into risk factors associated with greater chance of developing stroke in a specific population at Amazon forest. This study sought to explore the relationship between genetic markers of inflammatory process and the clinical risk of stroke in people with sickle cell anemia. The authors presented 70 patients (87.5%) were classified in group 1, without a stroke clinic, and 10 patients (12.5%) in group 2, with this clinic. These groups were subdivided in relation to the Doppler examination. Detailed analysis of genetic polymorphisms in specific subgroups revealed that variants in the *CYP19A1* and *MDM2* genes, in the group of patients without clinical stroke, showed statistical significance, indicating a possible protective effect against the stroke phenotype. Among patients who had a stroke, the variant in the *CASP8* gene was statistically significant, suggesting that some genotypes may have a greater effect on the evolution of the stroke in patients with sickle cell anemia. Future studies should seek to include more patients with complete Doppler data and explore other variables that may affect stroke risk.

## Introduction

Sickle cell anemia is a hemoglobinopathy characterized by the presence of hemoglobin S (HB S) in homozygosity. The World Health Organization (WHO) considers this disease as a public health problem projecting a worldwide increase in cases, by 2050, of approximately 405 thousand individuals (Díaz-Matallana et al., 2021; CDC, 2023).

In Brazil, estimates suggest that it may represent the most prevalent genetic disease. Around 4-8% of the population is black, with a higher incidence of the disease, but as ancestry is quite heterogenous in the country, the percentage of Sickle Cell Anemia trait carries increases the numbers of individuals with greater odds of developing symptoms from healthy carriers. It is estimated that 3,000 new cases of the disease each year. The numbers in this disease could affect 60,000 people in Brazil (Pinto et al., 2020). As it represents an important cause of stoke in childhood, the disease must be monitored based on genetic clues that corroborate the clinical outcome with cerebrovascular diseases.

The disease presents great phenotypic heterogeneity due to consequences of cell sickling that occurs in the microcirculation, in conditions of low oxygen tension or acidosis, where the deoxygenated HbS molecule polymerizes in erythrocytes, modifying their shape. This polymerization reduces the circulatory capacity of the erythrocyte which can cause vascular obstruction. After successive events of erythrocyte polymerization and depolymerization, various damages are caused until hemolysis occurs, generating the main clinical manifestations of the disease, such as vessel-occlusion and chronic hemolytic anemia (Ballas et al., 2010). 

With hemolysis, a decrease in the bioavailability of nitric oxide (NO) is generated, thus affecting vasodilatation and activation of the endothelium. Those events favor cell adhesion and consequent obstruction of the vessel, thus generating ischemia followed by reperfusion, when blood flow is restored. During ischemia tissue damage begins and with reperfusion, the inflammatory process begins. Theses successive ischemia and reperfusion events release inflammatory agents, increasing cell adhesion and further favoring vaso-occlusive events which it turns generates the characteristic severity of the disease, including stroke, the focus of this study (Debaun et al., 2020). 

These processes are mediated by a series of genes and their signaling pathways, making it essential to understand how genetic variants or mutations influence inflammation and its clinical consequences (Belisário et al., 2015; Boehme et al., 2017). 

Isolated cases of stroke show more significant risks for older people, but when associated with patients with Sickle cell anemia, the highest incidence of stroke occurs in younger people. In numbers, these cases appear predominantly in children and adolescents (5 - 17%) (Verduzco and Nathan, 2009, Kirkham and Lagunju, 2021).

Between the ages of 2 and 5, it affects 1.02% of children annually, with a consensus of low frequency up to 2 years of age - which can be explained by the influence of fetal hemoglobin, which restricts the events of morphological change in the hemoglobin molecule (Verduzco and Nathan, 2009, Kirkham and Lagunju, 2021). Around 11% of Sickle cell patients experience a stroke before turning 20, with this frequency increasing to 24% in the 4th decade of life (Verduzco and Nathan, 2009). 

Although the natural history of stroke associated with sickle cell anemia is well described, the pathophysiology of the event is still not fully understood (Connes et al., 2013). Apart from increased cerebral blood flow velocity detected by transcranial brain Doppler (TCD) (Adams et al., 1998), few risk factors are known. Belisário et al. (2015) showed the analysis of genetic biomarkers and the possibility of cerebrovascular disease occurring in a group of Brazilian children and estimated the risk of stroke at around 5.1% (± 3% - 7.2%).

Numerous biomarkers may be associated with clinical expression in sickle cell disease (SCD). Among them, the genetic ones that can be transmitted with the “Mendelian inheritance” through mutations and those through single nucleotide polymorphisms (SNP) through genes with different protein expressions than usual, such as those involved in vasomodeling processes such as angiotensin, HLA, as well as genes involved in the process of hypoxic inflammation and clot formation, stand out (Kirkham, 2007).

The research about many genes associated with the inflammatory process in patients with sickle cell anemia, including those related to stroke, is of great importance due to the central role of inflammation in the pathogenesis of this disease. The genes investigated here (*ACE, CAPS8, CYP2E1, CYP19A1, HLAG, IL1A, IL4, MDM2, NFKB1, SGSM3, TP53, TYMS, UGT1A1* and *XRCC1*) are involved in different aspects of the inflammatory response, from the modulation of cytokine expression and activity.to the regulation of cell death and DNA repair. For example, the *ACE* gene is related to the regulation of blood pressure and inflammation (Cozier et al., 2020) while *CASP8* is involved in apoptosis, an important process for the resolution of inflammation (Conus et al., 2008). The *NFKB1* pathway, in turn, is central in mediating the inflammatory response, influencing the expression of genes involved in immunity and inflammation (Mussbacher et al., 2019). Therefore, it is necessary to investigate this panel of inflammatory markers in the stroke clinic in patients with sickle cell anemia. 

## Subjects and Methods 

### Sample characterization

Eighty samples from patients with sickle cell anemia treated at the Fundação Centro de Hemoterapia e Hematologia do Pará (HEMOPA) were investigated, with ages ranging from 1 year to 22 years, with 58.83% of the patients being male.

The sample was divided into two groups, group 1 being patients without clinical stroke and group 2 patients with clinical stroke. Within these two groups, the samples were subdivided according to the results of the Doppler examination. 

Doppler examination is a method used for measuring the speed of cerebral blood flow, posing as an important tool to evaluate patients with clinical indications of strokes, vasospasms and embolies. Thus, in group 1 we have: (i) patients without stroke with Doppler above 170 cm/s, which suggests a conditional risk (based on clinical factors) and (ii) patients without stroke with normal Doppler (<170 cm/s), group 2: (iii) patients with stroke and Doppler above 170 cm/s and (iv) patients who had already had a stroke, with normal Doppler (<170 cm/s).

### Genotyping

The polymorphisms were genotyped by multiplex PCR reaction, using the QIAGEN Multiplex PCR Kit (Qiagen, Hilden, Germany). The protocol for the INDEL panel was standardized and first described by Marques et al. (2017). The multiplex PCR product was separated by capillary electrophoresis on the ABI3130 Genetic Analyzer, using GS-500 LIZ as molecular weight standard, G5 virtual filter matrix and POP7 (Thermo Fisher Scientific). Then, samples were analyzed with GeneMapper 3.7 software (Thermo Fisher Scientific).

### Data analysis

Comparisons between subgroups occurred for all 16 polymorphisms investigated in the panel. Differences in allele and genotype frequencies were measured using the chi-square test. Hardy-Weinberg equilibrium and Yates correction were performed, with p-value considered significant below 0.05. The statistical program used was JASP v.0.17.2.1 (JASP Team, 2024).

### Ethical issues

This study was submitted to the Research Ethics Committee involving Human Subjects (CAAE No. 64656316.0.0000.5169) and approved under protocol No. 1,941,530. Subsequently, an amendment was approved (protocol No. 4,928,767) to extend the study period and broaden the scope of data collection.

All participants provided written informed consent. For participants under 18 years of age, an Assent Form was obtained in addition to the Informed Consent Form signed by their legal guardians.

## Results

Seventy patients (87.5%) were classified in group 1, without a stroke clinic, and 10 patients (12.5%) in group 2, with clinical event of stroke.

Doppler Examination was performed in 37,5% of the total sample (30/80), only in patients who fit the clinical criteria for stroke risk. Group 1 was subdivided into 19 patients without stroke with normal Doppler (DN, 27.14%) and 5 patients without stroke with Doppler above 170cm/s, that is, Doppler at risk (RD, 7.14%). 46 patients in group 1 had no Doppler information (SI, 65.71%). Group 2 was subdivided into 3 patients with stroke and DN examination (30%) and 3 patients with stroke and DR (30%). Four patients in group 2 did not have information from the Doppler exam (40%) (Table 1). 

**Table 1 -:** Classification of patients with sickle cell anemia according to stroke phenotype and Doppler examination.

Sickle Cell Disease	Normal Doppler	Risk Doppler	No information
With stroke (n=10)	3 (30%)	3 (30%)	4 (40%)
Without stroke (n=70)	19 (27.14%)	5 (7.14%)	46 (65.71%)

In Table 2 it is possible to observe the demographic data of both groups. In our analyses, no statistical difference was found between the two groups for age, sex and two of the genomic ancestries studied (European and Native American). 

**Table 2 -:** Demographic data for group 1 (no stroke) and group 2 (stroke) of sickle cell anemia patients.

Variable	Without Stroke	With Stroke	P-value
N	70	10	
Age. years^a^	9.887±0.612	11.900±1.422	0.242
Sex. male/female	64.3/35.7	60.0/40.0	0.140
European	0.488±0.034	0.642±0.043	0.122
African	0.278±0.029	0.101±0.028	0.026
Native American	0.234±0.026	0.257±0.053	0.628

^a^ Values are expressed as mean ± SE (Standard Error of Mean). Student’s t-test.

Some samples did not amplify for some studied variants*.* Given the limited sample number, it was not possible to perform logistic regression for each genotype with adjustment considering the possible confounding factor (African ancestry, p=0.026). However, using chi-square with Yates correction as the general analysis, comparisons did not show statistically significant results for any of the genotypes.

The genotypic frequencies of samples from patients with sickle cell anemia without the stroke phenotype were compared with those of the group presenting this event for all investigated genetic variants, and no statistically significant differences were observed in any of the analyses (Table 3).

**Table 3 -:** Comparisons of genotype frequencies between groups of patients with sickle cell anemia and stroke phenotype.

Genes	Sickle cell disease		
	Without Stroke	With Stroke
ACE		
DEL/DEL	39	4
DEL/INS	19	5
INS/INS	9	1
P-value	0.386	
CASP8		
DEL/DEL	18	3
DEL/INS	23	5
INS/INS	28	2
P-value	0.426	
CYP2E1		
DEL/DEL	58	9
DEL/INS	6	1
INS/INS	1	0
P-value	0.923	
CYP19A1		
DEL/DEL	19	3
DEL/INS	30	5
INS/INS	21	2
P-value	0.806	
HLAG		
DEL/DEL	22	3
DEL/INS	42	7
INS/INS	5	0
P-value	0.653	
IL1A		
DEL/DEL	19	5
DEL/INS	34	1
INS/INS	17	4
P-value	0.070	
IL4		
DEL/DEL	9	3
DEL/INS	38	4
INS/INS	20	3
P-value	0.372	
MDM2		
DEL/DEL	8	2
DEL/INS	19	2
INS/INS	41	6
P-value	0.717	
NFKB1		
DEL/DEL	19	1
DEL/INS	36	6
INS/INS	13	3
P-value	0.432	
SGSM3		
DEL/DEL	18	1
DEL/INS	38	5
INS/INS	13	4
P-value	0.247	
TP53		
DEL/DEL	51	10
DEL/INS	13	0
INS/INS	6	0
P-value	0.169	
TYMS		
DEL/DEL	11	3
DEL/INS	31	5
INS/INS	27	2
P-value	0.388	
UGT1A1		
DEL/DEL	29	3
DEL/INS	30	6
INS/INS	10	1
P-value	0.618	
XRCC1		
DEL/DEL	7	0
DEL/INS	23	4
INS/INS	39	6
P-value	0.564	

Additional analyses were performed by subdividing Groups 1 and 2 according to the availability of Doppler examination results. The genotype frequencies of all studied variants are shown in Table 4. Analyzes were carried out using a dominant and recessive model, comparing the results of the homozygous genotypes (DEL/DEL or INS/INS) versus the other genotypes of the allele in question. The results were not statistically significant for the comparisons of the INS/INS genotype versus DEL/DEL homozygotes and DEL/INS heterozygotes. 

**Table 4 -:** Comparisons of genotype frequencies between subgroups of patients with sickle cell anemia stroke phenotype and Doppler examination results.

Genes	Sickle cell disease					
	Without Stroke			With Stroke		
	ND(%)	RD (%)	NI (%)	ND (%)	RD (%)	NI (%)
ACE						
DEL/DEL	11 (61.11%)	2 (40%)	26 (59.09%)	2 (66.66%)	1 (33.3%)	1 (25%)
DEL/INS	4 (22.22%)	3 (60%)	12 (27.27%)	1 (33.33%)	2 (66.66%)	2 (50%)
INS/INS	3 (16.67%)	0	6 (13.63%)	0	0	1 (25%)
P-value			0.533			0.453
CASP8						
DEL/DEL	6 (31.57%)	1 (20%)	11 (24.44%)	0	0	3 (75%)
DEL/INS	7 (36.84%)	1 (20%)	15 (33.33%)	3 (100%)	2 (66.66%)	0
INS/INS	6 (31.57%)	3 (60%)	19 (42.22%)	0	1 (33.33%)	1 (25%)
P-value			0.818			0.059
CYP2E1						
DEL/DEL	17 (94.44%)	4 (80%)	37 (88.09%)	3 (100%)	2 (66.66%)	4 (100%)
DEL/INS	1 (5.55%)	1 (20%)	4 (9.52%)	0	1 (33.33%)	0
INS/INS	0	0	1 (2.38%)	0	0	0
P-value			0.817			0.274
CYP19A1						
DEL/DEL	2 (10.5%)	0	17 (36.9%)	0	1 (33.33%)	2 (50%)
DEL/INS	11 (57.9%)	2 (40%)	17 (36.9%)	1 (33.33%)	2 (66.66%)	2 (50%)
INS/INS	6 (31.6%)	3 (60%)	12 (26.08%)	2 (66.66%)	0	0
P-value			0.089			0.168
HLAG						
DEL/DEL	5 (26.31%)	2 (40%)	15 (33.33%)	0	1 (33.33%)	2 (50%)
DEL/INS	13 (68.42%)	3 (60%)	26 (57.78%)	3 (100%)	2 (66.66%)	2 (50%)
INS/INS	1 (5.26%)	0	4 (8.89%)	0	0	0
P-value			0.878			0.358
IL1A						
DEL/DEL	5 (26.31%)	1 (20%)	13 (28.26%)	2 (66.66%)	2 (66.66%)	1 (25%)
DEL/INS	11 (57.89%)	2 (40%)	21 (45.65%)	0	0	1 (25%)
INS/INS	3 (15.78%)	2 (40%)	12 (26.08%)	1 (33.33%)	1 (33.33%)	2 (50%)
P-value			0.781			0.645
IL4						
DEL/DEL	2 (11.11%)	0	7 (15.99%)	2 (66.66%)	1 (33.33%)	0
DEL/INS	9 (50%)	4 (80%)	25 (56.81%)	0	2 (66.66%)	2 (50%)
INS/INS	7 (38.89%)	1 (20%)	12 (27.27%)	1 (33.33%)	0	2 (50%)
P-value			0.677			0.212
MDM2						
DEL/DEL	4 (22.22%)	2 (40%)	2 (4.44%)	1 (33.33%)	0	1 (25%)
DEL/INS	5 (27.77%)	1 (20%)	13 (28.89%)	0	0	2 (50%)
INS/INS	9 (50%)	2 (40%)	30 (66.67%)	2 (66.66%)	3 (100%)	1 (25%)
P-value			0.085			0.235
NFKB1						
DEL/DEL	6 (33.33%)	0	13 (28.89%)	0	1 (33.33%)	0
DEL/INS	9 (50%)	4 (80%)	23 (51.11%)	2 (66.66%)	2 (66.66%)	2 (50%)
INS/INS	3 (16.67%)	1 (20%)	9 (20%)	1 (33.33%)	0	2 (50%)
P-value			0.066			0.421
SGSM3						
DEL/DEL	7 (36.84%)	2 (40%)	9 (20%)	1 (33.33%)	0	0
DEL/INS	9 (47.36%)	2 (40%)	27 (60%)	2 (66.66%)	1 (33.33%)	2 (50%)
INS/INS	3 (15.78%)	1 (20%)	9 (20%)	0	2 (66.66%)	2 (50%)
P-value			0.628			0.343
TP53						
DEL/DEL	10 (52.6%)	2 (40%)	26 (56.52%)	1 (33.33%)	3 (100%)	3 (75%)
DEL/INS	8 (42.1%)	3 (60%)	17 (36.9%)	2 (66.66%)	0	0
INS/INS	1 (5.23%)	0	3 (6.5%)	0	0	1 (25%)
P-value			0.876			0.129
TYMS						
DEL/DEL	4 (22.22%)	0	7 (15.12%)	0	2 (66.66%)	1 (25%)
DEL/INS	6 (33.33%)	2 (40%)	23 (50%)	3 (100%)	1 (33.33%)	1 (25%)
INS/INS	8 (44.44%)	3 (60%)	16 (34.78%)	0	0	2 (50%)
P-value			0.559			0.114
UGT1A1						
DEL/DEL	9 (50%)	1 (20%)	19(41.3%)	1 (33.33%)	0	2 (50%)
DEL/INS	6 (33.33%)	3 (60%)	21 (45.65%)	2 (66.66%)	3 (100%)	1 (25%)
INS/INS	3 (16.67%)	1 (20%)	6 (13.04%)	0	0	1 (25%)
P-value			0.759			0.333
XRCC1						
DEL/DEL	1 (5.26%)	0	0	1 (33.33%)	0	0
DEL/INS	3 (15.78%)	2 (50%)	15 (32.69%)	0	0	1 (25%)
INS/INS	15 (78.94%)	2 (50%)	31 (67.39%)	2 (66.66%)	3 (100%)	3 (75%)
P-value			0.279			0.453

ND: Normal Doppler; RD: Risk Doppler e NI: No information

For comparisons of the DEL/DEL genotype versus INS/INS homozygotes and DEL/INS heterozygotes, statistically significant results were shown between the subgroups of patients in Group 1 (without stroke phenotype), in relation to the polymorphisms of the *CYP19A1* (rs11575899) (p=0.034) and *MDM2* (rs3730485) (p=0.018) genes, with higher genotype frequencies INS/INS and INS/DEL in any of the subgroups regardless of the test results (Table 5).

**Table 5 -:** Comparisons of DEL/DEL versus INS/INS and DEL/INS genotype frequencies of *CYP19A1* and *MDM2* genes in patients with sickle cell anemia in Group 1 subclassified according to transcranial Doppler examination.

Genes	Sickle cell disease		
	ND (%)	RD (%)	NI (%)
CYP19A1			
INS/DEL X INS/INS	17 (89.47%)	5 (100%)	29 (63.04%)
DEL/DEL	2 (10.52)	0	17 (36.95%)
P-value			0.034
MDM2			
INS/DEL X INS/INS	14 (77.78%)	3 (60%)	43 (95.56%)
DEL/DEL	4 (22.22%)	2 (40%)	2 (4.44%)
P-value			0.018

ND: Normal Doppler; RD: Risk Doppler e NI: No information.

A genotypic combination was carried out between these genetic variants and the risk characteristics classified in the sample. The results were not statistically significant, showing that these variants do not synergistically affect the risk of the stroke phenotype in this sample.

Comparisons in the subgroups of patients with stroke show that the DEL/DEL genotype - rs3834129 variant of the *CASP8* gene - presents statistically significant results (p=0.040), not being observed in the subgroups of patients with Doppler exams (Table 6).

**Table 6 -:** Comparisons of the genotype frequencies DEL/DEL versus INS/INS and DEL/INS of the *CASP8* gene in patients with sickle cell anemia in Group 2 with a stroke phenotype subclassified according to the transcranial doppler examination.

Genes	Sickle cell disease		
	ND (%)	RD (%)	NI (%)
CASP8			
INS/DEL x INS/INS	3 (100%)	3 (100%)	1 (25%)
DEL/DEL	0	0	3 (75%)
P-value			0.040

ND: Normal Doppler; RD: Risk Doppler e NI: No information.

## Discussion

The different forms of expression and levels of severity of sickle cell anemia in patients make clinical monitoring quite complex and necessary. Therefore, clinical studies that correlate genetics and clinical severity can be useful in informing the prognosis of diseases that, due to their potential manifestations, cannot be fully understood. 

Children and adolescents are more predisposed to the stroke phenotype in patients with sickle cell anemia, and there are few studies investigating the causes. Polymorphisms that influence the risk of stroke in children with sickle cell anemia, including variants in the *ANXA2, TGFBR3, TNF-α, IL6, ADCY9* and *TEK* genes were identified (Flanagan *et al*. 2011; Rafael et al., 2020; Scalise, 2023). These findings support the need to incorporate genetics into risk assessment for patients with sickle cell anemia, highlighting the importance of a personalized approach to disease management.

Detailed analysis of genetic polymorphisms in specific subgroups revealed that variants in the *CYP19A1* and *MDM2* genes, in the group of patients without clinical stroke, showed statistical significance, indicating a possible protective effect against the stroke phenotype in patients with the INS allele in homozygosity or heterozygosity for the two variants analyzed. Among patients who had a stroke, the variant in the *CASP8* gene was statistically significant, suggesting that the INS/INS and INS/DEL genotypes may have a greater effect on the evolution of the stroke in patients with sickle cell anemia.

The *CYP19A1* gene encodes the aromatase enzyme, which is involved in the biosynthesis of estrogens from androgens, so changes in the activity of this enzyme can influence hormonal balance and have diverse effects on physiological and pathological processes, including the regulation of vascular function and inflammation (Baghaei et al., 2003; Dunning et al., 2004; Tworoger et al., 2004). Haiman et al. (2000) and Tworoger *et al*. (2004) suggest that the rs11575899 deletion/insertion polymorphism may act by binding with greater affinity to proteins that reduce the production of androgens and all hormone biosynthesis, which would culminate in a greater risk of vaso-occlusive events.

Our results show that the presence of the repeat (INS allele) whether in homozygosity or heterozygosity would be at higher frequencies in groups of patients with sickle cell anemia who do not have the stroke phenotype, suggesting that they may be reducing the risk of inflammatory processes that would lead to vessel occlusion.

The *MDM2* gene is an important regulator of the *TP53* gene, which acts as a “guardian of the genome” and actively participates in the apoptosis of cells with changes in the genetic material. Miedl et al. (2020) show that the rs3730485 (INS/DEL) polymorphism impacts the regulation of *TP53* gene expression, being inhibited when the DEL allele is homozygous.

Our statistically significant results showed the lowest frequencies of this homozygous DEL/DEL genotype in all subgroups of patients with sickle cell anemia, without stroke phenotype. This may suggest that the MDM2 gene is functioning properly and preventing other genes, dependent on its function, from dysregulating and influencing vascular homeostasis, culminating in vaso-occlusive episodes that increase the risk of the stroke phenotype.

Regarding the results in the group of patients with sickle cell anemia and stroke phenotype, the results were statistically significant only when comparing the DEL/DEL genotypes versus the other associated genotypes. The *CASP8* gene acts in the production of an important protein from the caspase family, which participates in cell apoptosis. The rs3834129 (INS/DEL) polymorphism has been investigated affecting this apoptotic pathway. Sun et al. (2007) show that the deletion (DEL allele) removes binding to the transcription factor and thus decreases cellular apoptosis events.

A possible hypothesis for our results showing low frequencies of the DEL/DEL genotype in samples from patients with sickle cell anemia who had a stroke phenotype may be related to the fact that if this genotype were present, there would probably be a decrease in apoptosis and a lower risk of vessel occlusion. Therefore, our studies suggest a possible protective effect of this genotype against risk factors that affect the stroke phenotype in patients with sickle cell anemia.

There is great phenotypic heterogeneity in patients with sickle cell anemia, related to genetic and environmental risk factors. The use of tests such as Doppler allows for a more detailed analysis of stroke risk and accelerates the possibilities of treatment and prevention of the severity of these phenotypes. Investigating subgroups of patients classified according to risk tests and clinical severity may favor clearer observations about genetic associations in more homogeneous subgroups.

Although all patients with sickle cell anemia treated at HEMOPA who presented with stroke as a clinical manifestation are included in this sample, the study is limited by the small sample size and, therefore, the observed associations should be interpreted with caution due to the risk of false positives. Furthermore, the absence of Doppler information in a significant proportion of patients restricts more precise analyses and may obscure relevant associations. Future studies should seek to include more patients with complete Doppler data and explore other variables that may affect stroke risk. 

Despite these limitations, given the scarcity of literature on the subject, our study explored for the first time the association between this panel of variants in genes of pathways related to inflammation and apoptosis with the stroke phenotype in sickle cell anemia, highlighting the importance of investigating this condition.

Another limitation of the present study concerns the absence of comparative analyses between hematological variables and the transfusion and pharmacological therapies adopted in the patients evaluated. Although hematological parameters are widely used to assess clinical progression and therapeutic response, these indicators can be directly influenced by interventions such as the use of hydroxyurea and blood transfusions, thus limiting their suitability for comparisons between different groups of patients (Ware and Aygun, 2009; Chou, 2013; Steinberg et al., 2014). In this context, the use of other statistical analyses with adjusted analytical models capable of simultaneously incorporating hematological variables and different therapeutic regimens, in order to minimize methodological bias, may increase the scientific accuracy and clinical relevance of the findings. This approach will help reduce potential misinterpretations and strengthen the robustness of the conclusions.

## Supplementary material

The following online material is available for this article:

Table S1 - Stroke phenotype, Doppler examination and genotype of patients with sickle cell anemia in the present study.

## Data Availability

 The data used in this study are available in Supplementary Table 1.
