# Supplementary material for: Relationship between biomarkers and stroke risk in sickle cell disease odds in an Amazon folk
Source: Genet Mol Biol. 2026 Jul 10;49(Suppl 4):e20250107. doi: 10.1590/1678-4685-GMB-2025-0107 (PMC13383251; doi:10.1590/1678-4685-GMB-2025-0107)
Supplement: Table S1 - [file 1415-4757-GMB-49-s4-e20250107-s1.pdf]

Genetics and Molecular Biology
https://doi.org/10.1590/1678-4685-GMB-2025-0107

Supplementary Material to: "Relationship between biomarkers and stroke risk in sickle cell disease odds in an amazon folk"

Table S1: Stroke phenotype, Doppler examination and genotype of patients with sickle cell anemia in the present study.

ID	DOPPLER[1]	Stroke[2]	*CYP19A1*	*NFKB1*	*TYMS*	*IL1A*	*CASP8*	*SGSM3*	*UGT1A1*	*TP53*	*MDM2*	*IL4*	*ACE*	*HLAG*	*XRCC1*	*CYP2E1*
VAF01	ND	0	D/I	D/I	D/D	D/I	I/I	D/I	D/D	D/D	D/D	I/I	D/D	D/I	I/I	D/D
VAF03	ND	0	D/I	D/I	D/I	D/D	D/I	D/I	D/D	D/I	D/D	D/I	D/D	D/D	I/I	D/D
VAF05	NI	0	D/I	D/D	D/D	D/I	D/I	D/I	D/D	D/D	I/I	D/I	D/D	D/I	D/I	D/D
VAF06	NI	0	I/I	I/I	D/I	D/I	I/I	NI	D/I	D/D	I/I	D/I	NI	D/I	D/I	D/D
VAF07	NI	0	D/I	D/I	D/D	I/I	D/D	D/D	D/I	D/D	NI	D/D	D/D	D/I	I/I	NI
VAF08	NI	0	D/D	D/D	D/I	D/I	D/I	I/I	D/D	D/D	D/D	D/I	D/D	D/I	D/I	D/D
VAF09	NI	0	D/I	D/I	I/I	D/I	I/I	I/I	D/I	I/I	D/I	D/I	I/I	D/D	I/I	D/D
VAF10	NI	0	D/D	D/D	D/I	D/I	D/D	D/I	D/I	D/D	D/I	D/I	D/I	D/D	I/I	D/D
VAF11	NI	0	D/I	D/D	D/D	D/D	D/I	I/I	D/I	D/D	D/D	D/I	D/D	D/D	D/I	D/D
VAF12	ND	0	I/I	I/I	I/I	I/I	D/I	D/I	I/I	D/D	I/I	D/I	D/D	D/I	D/I	D/D
VAF13	ND	0	D/I	D/I	D/I	D/I	D/D	D/D	D/D	D/D	D/I	D/D	D/D	D/D	D/D	D/D
VAF14	ND	0	D/I	D/D	I/I	D/D	D/D	I/I	D/D	D/I	D/I	D/I	D/D	D/I	I/I	D/D
VAF15	ND	0	I/I	D/I	I/I	D/D	D/I	D/D	I/I	D/I	I/I	D/I	D/D	D/D	I/I	D/D
VAF16	NI	0	D/D	I/I	D/I	D/D	I/I	D/I	D/D	D/D	I/I	D/I	D/D	D/I	D/I	D/D
VAF17	ND	0	I/I	D/I	I/I	D/I	D/I	D/I	D/D	D/D	I/I	D/I	D/I	D/I	D/I	D/D
VAF18	NI	1	D/D	I/I	I/I	I/I	D/D	D/I	D/I	D/D	D/D	D/I	D/I	D/I	I/I	D/D
VAF19	ND	0	I/I	D/I	I/I	D/I	I/I	D/I	D/I	D/D	D/I	D/I	D/D	D/I	I/I	D/I
VAF20	NI	0	I/I	D/I	I/I	D/D	I/I	D/D	I/I	D/D	I/I	D/I	D/I	D/I	D/I	D/D
VAF21	RD	0	D/I	D/I	I/I	I/I	D/D	D/D	I/I	D/D	I/I	D/I	D/I	D/I	I/I	D/D
VAF22	RD	1	D/D	D/I	D/D	I/I	I/I	D/I	D/I	D/D	I/I	D/D	D/I	D/I	D/I	D/I
VAF23	ND	0	D/I	D/I	I/I	D/I	D/I	D/D	D/I	D/D	D/I	D/I	D/I	D/D	I/I	D/D
VAF24	NI	0	I/I	D/I	I/I	D/I	I/I	D/I	I/I	D/D	I/I	I/I	D/D	D/I	I/I	D/I
VAF25	NI	0	D/D	D/I	I/I	I/I	D/I	D/I	D/I	D/D	D/I	D/D	D/D	D/I	I/I	D/D

ID	DOPPLER[1]	Stroke[2]	*CYP19A1*	*NFKB1*	*TYMS*	*IL1A*	*CASP8*	*SGSM3*	*UGT1A1*	*TP53*	*MDM2*	*IL4*	*ACE*	*HLAG*	*XRCC1*	*CYP2E1*
VAF26	NI	0	D/D	D/I	D/I	I/I	I/I	D/I	D/I	D/D	I/I	D/I	D/D	D/I	D/I	D/D
VAF27	ND	0	D/D	D/I	D/I	I/I	D/D	D/D	D/D	D/D	I/I	D/I	D/D	D/I	D/I	D/D
VAF29	NI	0	D/I	D/I	D/I	D/D	I/I	D/I	I/I	D/D	I/I	D/I	D/I	D/I	D/D	D/D
VAF30	NI	0	D/D	I/I	I/I	D/D	I/I	D/D	D/I	D/D	I/I	I/I	D/I	D/D	D/I	D/D
VAF31	NI	0	D/D	D/I	D/I	I/I	D/I	D/I	I/I	D/D	I/I	I/I	D/D	D/D	D/D	D/D
VAF32	ND	0	D/D	D/I	D/D	D/I	D/I	D/I	D/I	D/I	D/D	I/I	D/D	D/I	D/D	D/D
VAF33	ND	1	I/I	D/I	D/I	I/I	D/I	D/I	D/I	D/D	I/I	D/D	D/D	D/I	D/I	D/D
VAF34	NI	0	I/I	D/I	D/I	D/D	D/D	D/I	D/I	D/D	I/I	D/I	D/I	D/I	I/I	D/I
VAF35	RD	0	D/I	D/I	D/I	I/I	I/I	D/I	D/I	D/I	I/I	D/I	D/I	D/D	D/D	D/D
VAF36	NI	0	I/I	NI	D/D	I/I	NI	D/I	D/D	D/D	I/I	NI	NI	D/D	I/I	D/D
VAF38	NI	0	D/D	D/D	I/I	I/I	D/I	I/I	D/D	I/I	I/I	D/I	D/D	D/I	D/I	D/I
VAF39	NI	0	D/D	D/D	D/I	D/I	D/I	D/I	D/D	D/D	D/I	D/D	D/D	D/D	I/I	D/D
VAF40	NI	0	D/I	D/I	D/D	D/I	I/I	I/I	D/D	D/D	I/I	D/I	D/I	D/I	D/D	D/D
VAF41	NI	0	D/D	I/I	I/I	D/D	I/I	D/I	I/I	D/I	I/I	NI	I/I	I/I	I/I	NI
VAF44	NI	0	D/I	D/D	I/I	D/D	D/I	D/D	D/I	I/I	I/I	I/I	D/D	D/I	I/I	D/D
VAF45	ND	0	I/I	D/D	D/I	D/D	I/I	D/D	I/I	I/I	I/I	I/I	D/D	D/I	I/I	D/D
VAF48	ND	1	D/I	I/I	D/I	D/D	D/I	D/D	D/D	D/D	I/I	D/D	D/I	D/I	I/I	D/D
VAF49	ND	0	I/I	D/D	I/I	D/I	D/I	D/I	D/I	D/D	I/I	I/I	D/I	D/I	I/I	D/I
VAF50	NI	1	D/I	D/I	I/I	D/D	D/D	I/I	I/I	D/D	D/I	D/I	D/D	D/I	I/I	D/D
VAF51	ND	0	I/I	D/D	D/I	D/I	I/I	D/I	D/D	D/D	I/I	D/I	I/I	D/I	D/I	D/D
VAF52	NI	0	I/I	D/I	D/I	D/I	D/D	I/I	D/I	D/D	I/I	D/I	D/I	D/I	I/I	D/D
VAF53	NI	0	D/D	D/D	I/I	D/I	D/D	D/I	D/I	D/I	I/I	D/I	D/D	D/D	I/I	D/D
VAF56	NI	0	D/D	D/D	I/I	D/D	I/I	D/D	D/I	D/D	I/I	D/I	D/I	D/I	I/I	D/D
VAF57	NI	0	D/I	D/I	D/I	D/D	D/I	D/I	D/D	D/D	I/I	I/I	I/I	D/D	I/I	D/D
VAF58	ND	1	D/I	D/I	D/D	D/D	D/I	I/I	D/I	D/D	I/I	D/I	D/D	D/D	D/I	D/D
VAF59	NI	0	D/I	I/I	D/I	D/I	D/D	D/D	D/I	D/D	D/I	D/I	D/I	D/D	I/I	D/D
VAF60	NI	0	D/I	D/I	D/I	D/I	I/I	D/I	D/D	D/D	I/I	D/I	I/I	D/I	D/I	D/D
VAF62	NI	0	D/I	D/D	D/I	D/D	I/I	I/I	D/D	D/D	D/I	D/D	I/I	I/I	I/I	D/I
VAF65	NI	0	D/I	I/I	D/I	D/D	I/I	D/D	D/D	D/I	I/I	I/I	D/D	D/I	D/I	D/D
VAF66	NI	0	I/I	D/I	D/I	D/I	I/I	D/I	D/D	D/I	D/I	I/I	D/D	I/I	I/I	D/D
VAF67	ND	0	D/I	I/I	D/D	D/I	I/I	I/I	D/D	D/D	D/D	I/I	D/I	D/I	D/I	D/D

ID	DOPPLER[1]	Stroke[2]	*CYP19A1*	*NFKB1*	*TYMS*	*IL1A*	*CASP8*	*SGSM3*	*UGT1A1*	*TP53*	*MDM2*	*IL4*	*ACE*	*HLAG*	*XRCC1*	*CYP2E1*
VAF68	NI	0	D/D	D/I	I/I	D/I	D/D	D/I	D/I	D/D	I/I	I/I	D/D	D/I	D/D	D/D
VAF70	NI	0	I/I	D/I	I/I	D/I	D/D	I/I	D/I	D/D	I/I	D/D	D/D	D/D	I/I	D/D
VAF72	RD	0	I/I	D/I	D/I	D/D	I/I	D/D	D/I	D/D	D/D	I/I	D/D	D/D	I/I	D/D
VAF73	NI	0	D/I	D/I	I/I	I/I	I/I	D/I	D/D	D/D	D/I	I/I	I/I	D/I	I/I	D/D
VAF74	NI	1	D/I	D/I	D/I	I/I	I/I	I/I	D/D	D/D	D/I	I/I	D/I	D/D	I/I	D/D
VAF76	ND	0	D/I	D/I	D/D	D/I	D/D	D/I	D/I	D/D	D/I	I/I	D/D	D/I	I/I	D/D
VAF77	ND	0	D/I	I/I	I/I	D/I	I/I	D/D	D/D	D/I	I/I	I/I	D/D	D/I	D/I	D/D
VAF79	RD	1	D/I	D/D	D/I	D/D	D/I	I/I	D/I	D/D	I/I	D/I	D/I	D/I	I/I	D/D
VAF81	NI	0	D/I	D/D	I/I	I/I	D/I	D/D	D/I	D/D	I/I	D/I	D/D	D/D	I/I	D/D
VAF82	ND	0	D/I	D/D	D/I	D/I	D/D	D/D	D/I	D/D	I/I	D/I	I/I	I/I	I/I	D/D
VAF83	ND	1	I/I	D/I	D/I	D/D	D/I	D/I	D/I	D/D	D/D	I/I	D/D	D/I	I/I	D/D
VAF84	ND	0	D/I	D/D	I/I	I/I	I/I	D/I	D/I	D/D	D/I	D/D	I/I	D/I	I/I	D/D
VAF85	NI	1	D/D	I/I	D/D	D/I	D/D	D/I	D/D	D/D	I/I	I/I	I/I	D/D	D/I	D/D
VAF86	NI	0	D/I	D/D	I/I	D/I	D/I	I/I	D/D	D/D	I/I	D/I	D/D	NI	I/I	NI
VAF87	NI	0	I/I	I/I	D/I	I/I	D/D	D/I	D/I	D/D	I/I	I/I	D/D	D/I	I/I	NI
VAF88	NI	0	I/I	I/I	D/D	D/I	D/D	D/I	D/I	D/D	I/I	D/I	D/D	I/I	D/I	I/I
VAF89	NI	0	D/I	D/I	D/I	D/D	I/I	D/I	D/I	D/D	D/I	D/I	D/D	D/I	I/I	D/D
VAF90	NI	0	I/I	D/I	D/I	I/I	D/I	D/I	D/D	D/D	I/I	D/I	D/D	D/I	D/I	D/D
VAF92	NI	0	D/D	D/I	D/I	D/I	I/I	D/I	D/D	D/D	D/I	D/I	D/I	D/D	I/I	D/D
VAF93	NI	0	D/D	D/I	I/I	D/I	D/I	D/I	D/D	D/I	D/I	D/I	D/I	D/D	D/I	D/D
VAF94	RD	0	I/I	I/I	I/I	D/I	D/I	I/I	D/D	D/D	D/D	D/I	D/I	D/I	D/I	D/D
VAF95	NI	0	D/I	D/I	D/D	I/I	D/I	D/I	D/D	D/I	D/I	D/D	D/D	D/I	D/I	D/D
VAF96	ND	0	D/I	NI	NI	D/D	D/D	I/I	NI	I/I	NI	NI	NI	D/D	NI	NI
VAF97	NI	0	I/I	I/I	D/I	D/I	I/I	D/I	D/I	D/I	D/I	I/I	D/I	D/I	D/I	D/D
VAF98	NI	0	D/D	D/I	D/I	D/I	D/I	D/D	I/I	I/I	I/I	D/D	D/D	D/D	I/I	D/D
VAF99	NI	0	D/D	D/D	D/I	I/I	D/D	D/I	D/D	D/D	I/I	I/I	D/D	D/I	I/I	D/D

[1]: ND: Normal Doppler; RD: Risk Doppler. [2]: 0: Ausent Stroke; 1: Present Stroke. Genotypes: D/D: homozygosity deletion; I/I: homozygosity insertion, D/I: heterozygosity deletion and insertion. NI: No information.
